# Breast sensibility after mastectomy and implant-based breast reconstruction

**DOI:** 10.1007/s10549-019-05137-8

**Published:** 2019-02-12

**Authors:** E. Bijkerk, S. M. J. van Kuijk, J. Beugels, A. J. M. Cornelissen, E. M. Heuts, R. R. W. J. van der Hulst, S. M. H. Tuinder

**Affiliations:** 1 Department of Plastic and Reconstructive Surgery, Maastricht University Medical Center, Maastricht, the Netherlands; 2 Department of Clinical Epidemiology and Medical Technology Assessment, Maastricht University Medical Center, Maastricht, the Netherlands; 3 Department of Surgery, Maastricht University Medical Center, Maastricht, the Netherlands

**Keywords:** Sensibility, Breast cancer, Alloplastic breast reconstruction, Mastectomy, Implant-based breast reconstruction

## Abstract

**Purpose:**

The aim of the study is to evaluate the level of sensible impairment after mastectomy or implant-based breast reconstruction (IBBR). In addition, factors influencing breast sensibility were evaluated.

**Methods:**

A cross-sectional study was performed in Maastricht University Medical Center between July 2016 and August 2018. Women with unilateral mastectomy with or without IBBR were included. Objective sensory measurements were performed using Semmes–Weinstein monofilaments. Their healthy breast served as control, using a paired *t* test. Differences between mastectomy with and without IBBR were evaluated using the independent *t* test. Linear regression was performed to evaluate the association between patient characteristics on breast sensibility. The paired *t* test was used to evaluate in which part of the breast the sensibility is best preserved.

**Results:**

Fifty-one patients were eligible for inclusion. Sixteen patients underwent IBBR after mastectomy. Twenty-three patients received radiotherapy and 35 patients received chemotherapy. Monofilament values were significantly higher in the operated group compared to the reference group (*p* < 0.001). Linear regression showed a statistically significant association between IBBR and objectively measured impaired sensation (*p* = 0.008). After mastectomy, the cutaneous protective sensation is only diminished. After IBBR, it is lost in the majority of the breast. The medial part of the breast was significantly more sensitive than the lateral part in all operated breasts (*p* < 0.001).

**Conclusion:**

IBBR has a significantly negative impact on the breast sensibility compared to mastectomy alone. This study shows that the protective sensation of the skin in the breast is lost after IBBR. To our knowledge, this is the first study to evaluate the level of sensible impairment after mastectomy or IBBR. More research is necessary to confirm these results.

**Electronic supplementary material:**

The online version of this article (10.1007/s10549-019-05137-8) contains supplementary material, which is available to authorized users.

## Introduction

Because survival rates of breast cancer have increased over the past years, more women have to live with the consequences of breast cancer treatment, often affecting the quality of life (QoL). When diagnosed with breast cancer, women face a variety of options concerning their treatment. Breast-conserving therapy (BCT) and radical mastectomy are a major part of breast cancer treatment. In the Netherlands, mastectomy is performed in 33–40% of breast cancer patients. Only 20% of these women desire reconstruction of the amputated breast, either immediate (26%) or delayed (74%) [1, 2], using their own tissue or prosthesis. Although autologous reconstruction has become standard care for breast cancer patients, implant-based breast reconstruction (IBBR) is still widely used. In the Netherlands, over 11 000 implants have been used for reconstructive purposes in 2017 [2]. The choice of undergoing reconstructive surgery is a very personal one. For making this decision, many factors have to be taken into consideration. One of those factors is the sensory change of the breast after mastectomy and reconstruction.

The primary goal of breast reconstructive surgery is to improve the quality of life (QoL) for breast cancer patients. Breast sensibility has not always been a priority, but it has become more important since better breast sensibility is associated with a higher QoL [3]. Preservation of breast sensibility is therefore desirable. Sensation of the breast is characterized by different aspects of tactile receptors (touch and pressure sensitivity), thermic receptors (heat and cold), pruriceptors (itch), and nociceptors (pain) [4]. In addition, the feeling of the breast is complicated by erogenous sensation.

Little is known about what patient characteristics or her treatment are associated with sensibility of the reconstructed breast. Therefore, the aim of the current study was to evaluate the consequences of mastectomy and IBBR on breast sensibility in women who underwent unilateral mastectomy for oncological purposes. Additional paired analysis was performed to evaluate in which part of the breast the sensibility is best preserved after mastectomy, with or without IBBR. To our knowledge, this is the first study to evaluate the level of sensible impairment after mastectomy with or without IBBR.

## Materials and methods

The STROBE statement for observational studies [5] was used for reporting this article. The study was carried out in compliance with the world medical association Declaration of Helsinki (2013) [6]. Ethical approval was obtained by the local Medical Ethical Committee (METC) of Maastricht University.

### Study population

This cross-sectional study was conducted in the Maastricht University Medical Center in Maastricht, the Netherlands between July 2016 and August 2018. The study population consisted of female breast cancer patients of 18 years or older who underwent unilateral mastectomy, with or without immediate implant-based reconstruction. Demographic data of each patient were collected. Written informed consent was obtained from each patient included in the study. Exclusion criteria were sensory measurement could not be performed, bilateral breast cancer and known neurological conditions that could affect the sensibility, such as diabetic neuropathy. Peripheral polyneuropathy due to chemotherapy or radiotherapy was not considered to be an exclusion criterion.

### Sensory testing

Sensation measurements were performed using Semmes–Weinstein monofilaments. In each patient, the cutaneous threshold in the breast region was measured using a 20-piece kit Semmes–Weinstein monofilaments, with index values ranging from 1.65 (thinnest monofilament) to 6.65 (thickest monofilament). Each index value represents the logarithm of the force in milligrams required to bend the monofilament into a C-shape. Therefore, a thinner monofilament requires less pressure to bend and corresponds to better sensation. Perpendicular pressure was applied to the same spot for a duration of 1.5 s, three times in succession. Testing started with the thinnest monofilament and proceeded in ascending fashion until touch was identified by the patient. Patients were in supine position and were asked to close their eyes. The different sites were tested in a random sequence.

According to the manufacturer’s instructions, the sensibility can be clustered into 1 of 5 levels, each marked a color on the rods. Green represents normal touch, blue diminished light touch, purple diminished protective sensation, and red loss of protective sensation or deep pressure sensation only [7].

### Areas measured

**Fig. 1:**
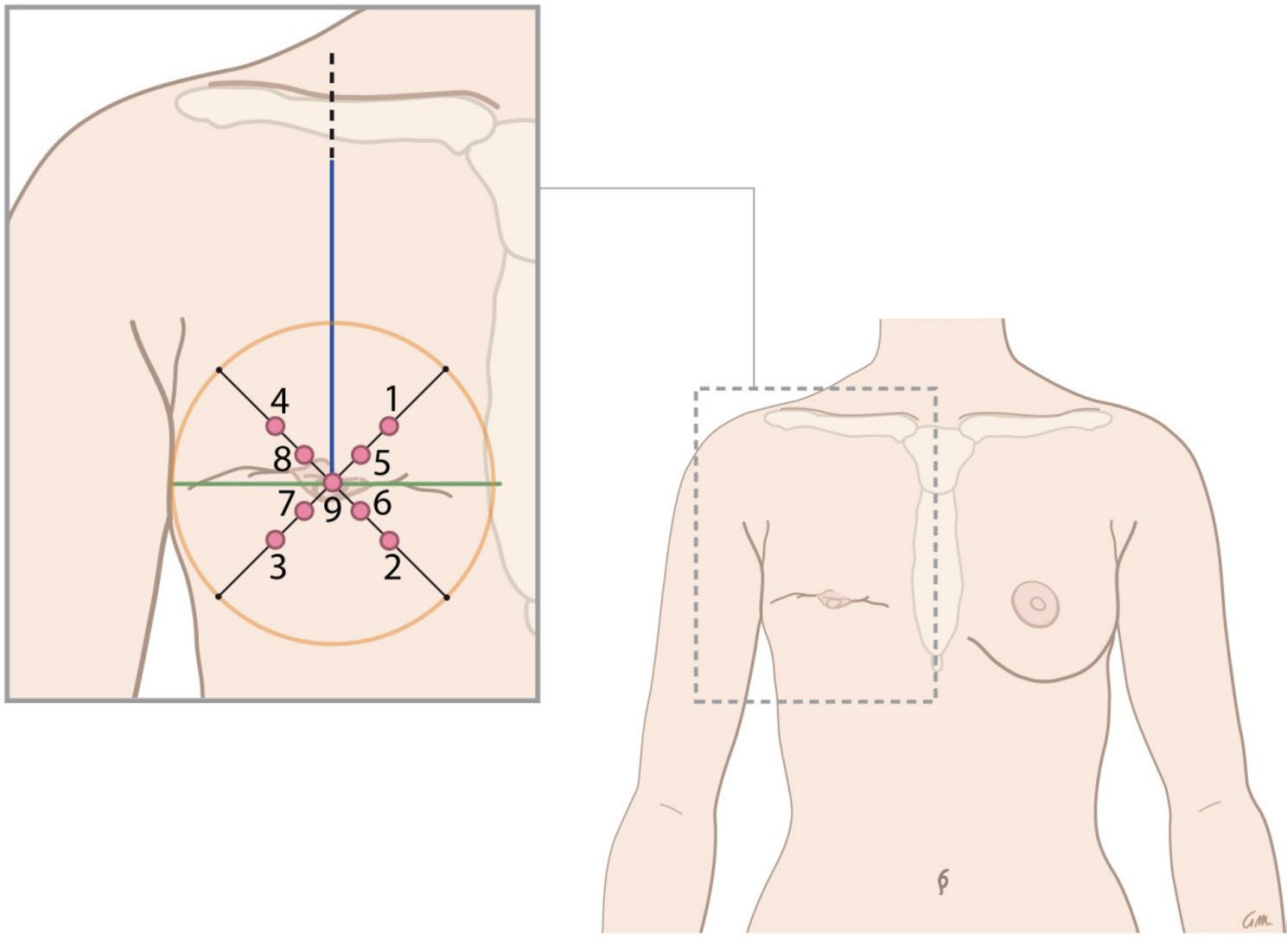
Areas measured according to the anatomical landmarks of the breast. Anatomical references of the non-operated, healthy breast on the left side of the patient and the mastectomy on the right side of the patient with a heat map of measurement points. The measured points were numbered: 1 = superomedial, 2 = inferomedial, 3 = inferolateral, 4 = superolateral, 5 = areola superomedial, 6 = areola inferomedial, 7 = areola inferolateral, 8 = areola superolateral, and 9 = nipple

Anatomical references were used to divide the breast surface into nine areas to be measured. In patients who had undergone mastectomy without reconstruction, two lines divided the breast area into four quadrants: one vertical line from midclavicular to caudal and one horizontal line perpendicular to the first. The lines crossed at the level of the contralateral nipple and the intersection (where the nipple used to be) formed the center of the circle drawn around the previous borders of the breast tissue. In patients who underwent immediate implant-based reconstruction, the inframammary and supramammary creases of the breast was determined according to the maneuver described by Marchac and de Olarte [8]. Each quadrant was divided by 45° lines. Figure 1 shows the nine areas corresponding to the normal breast and the mastectomy side. Area 1 to 4 were halfway on each of the 45° lines; 5 to 8 were in the areola in each quadrant; and area 9 was located on the nipple or where it was supposed to be.

### Statistical analysis

Because little was known before the start of the study that could be used to calculate a necessary sample size, we chose a pragmatic approach. All available patients that fit the eligibility criteria were asked to participate.

Each woman contributed two observations to the data: the operated breast and the contralateral, non-mastectomy breast as a control. After that, we subdivided operated breasts into those that underwent mastectomy alone and those who received implant-based breast reconstruction after mastectomy. Since both the operated and control breasts are from the same sample of women, baseline characteristics are identical between the groups of observations. Continuous variables were reported as mean and standard deviation (SD) or median and interquartile range (IQR), depending on the distribution of the variables. Categorical variables were reported as absolute numbers and percentages. Continuous variables were compared using the paired-samples t test or the Wilcoxon signed-rank test, and categorical variables were tested using the McNemar test. In case of missing data, stochastic regression imputation was used to complete the dataset to prevent loss of statistical precision and decrease the likelihood of bias.

The primary outcome was assessed as the difference in sensation between the operated breasts and the contralateral non-operated breasts. The median of all sensation measurements per breast was taken as a summary of sensibility of the breast. The paired-samples *t* test was used to evaluate the differences in breast sensibility between all operated breasts and their references, between the breasts that only underwent mastectomy and their references, and between the breasts that underwent implant-based breast reconstruction after mastectomy and their references. Simple and multiple linear regression analyses were used to estimate the crude and adjusted associations between patient characteristics and the difference in sensation between the operated and control breast. The independent samples *t* test was used to evaluate the differences in sensation between the breasts with an implant in situ and the operated breasts that underwent mastectomy alone.

To evaluate in which part of the breast the sensibility is best preserved, a paired-samples *t* test was used. In all breasts the medial part was defined as the mean of the measurements of area 1, 2, 5, and 6; the lateral part was defined as the mean of the measurements of area 3, 4, 7, and 8; the cranial part was defined as the mean of the measurements of area 1, 4, 5, and 8; and the caudal part was defined as the mean of the measurements of area 2, 3, 6, and 7. Subgroup analysis was performed between the quadrants. The upper inner quadrant (UIQ) was the mean of measurements of area 1 and 5; the lower inner quadrant (LIQ) as the mean of area 2 and 6; the upper outer quadrant (UOQ) was defined as the mean of measurements of area 4 and 8; and the lower outer quadrant (LOQ) as the mean of area 3 and 7.

A *p* value < 0.05 was considered as statistically significant. All analyses were performed using IBM SPSS for Windows (Version 23.0, Released 2015. Armonk, NY: IBM Corp).

## Results

### Patient characteristics

A total of 55 patients came to Maastricht University Medical Center for a unilateral secondary or tertiary autologous breast reconstruction between July 2016 and August 2018, of which 51 patients were eligible for inclusion. Three patients were known with diabetes mellitus, one patient had a wound at the mastectomy side and therefore sensory measurements could not be performed. All included women previously underwent unilateral mastectomy due to breast cancer treatment, with or without implant-based breast reconstruction. The mean age was 53 years (range 33–69) and the mean BMI was 25.1 (range 19.84–31.64). A total of 23 patients (45.1%) received adjuvant or neo-adjuvant radiotherapy and 35 patients (68.6%) chemotherapy. In 16 patients (31.4%), who previously underwent breast augmentation or implant-based reconstruction after mastectomy, the prosthesis was still in situ. Five patients (9.8%) had undergone surgery on the contralateral breast before sensory measurements, of which four patients had a contralateral reduction for symmetry after previous breast cancer surgery. Only one patient underwent augmentation of her breasts before she was diagnosed with breast cancer and also had an implant in the contralateral, non-mastectomy breast. The median time between the mastectomy and the objective sensory measurements was 22.0 months (IQR 15.0–61.0). None of the patients smoked at the time of the sensory measurements.

### Sensation in the operated breast

Median monofilament values with the interquartile range for each area of the all operated breasts and the reference group (*n* = 51) are summarized in Table 1. The total median monofilament index value of the operated breast was 4.17 (IQR 3.22–4.93).This was significantly higher than the median monofilament index value of the reference group, which was 2.36 (IQR 2.36–2.83), indicating less sensibility of the operated breast. This applied to all the areas measured independently, as well as the sensation measured in the total breast (Table 1).

**Table 1:** Median monofilament index values per area and in total of all breasts

Mean monofilament index value	Reference (*n* = 51)	Operated breast (*n* = 51)	*p *value
1. Upper inner quadrant (UIQ)	2.36 (2.36–2.44)	3.61 (2.36–4.31)	< *0.001*
2. Lower inner quadrant (LIQ)	2.36 (2.36–2.83)	3.61 (2.36–4.31)	< *0.001*
3. Lower outer quadrant (LOQ)	2.36 (2.36–2.83)	3.84 (2.83–4.31)	< *0.001*
4. Upper outer quadrant (UOQ)	2.36 (2.36–2.44)	3.61 (2.83–4.56)	< *0.001*
5. Areola upper inner	2.36 (2.36–2.83)	4.31 (3.22–4.93)	< *0.001*
6. Areola lower inner	2.44 (2.36–3.22)	4.31 (3.22–5.18)	< *0.001*
7. Areola lower outer	2.44 (2.36–3.22)	4.31 (3.84–5.18)	< *0.001*
8. Areola upper outer	2.36 (2.36–3.22)	4.31 (3.84–5.46)	< *0.001*
9. Nipple	2.83 (2.36–3.22)	4.56 (4.08–5.46)	< *0.001*
10. Total breast	2.36 (2.36–2.83)	4.17 (3.22–4.93)	< *0.001*

Data are shown as median with interquartile range (IQR)

Statistically significant differences are indicated in italic

### Subgroup analysis

After subdividing the total group into the alloplastic reconstructed breasts (*n* = 16) and the mastectomies (*n* = 35), patient characteristics between both groups were compared, as shown in Table 2. The groups differed significantly in the number of previous breast surgeries, time between mastectomy and sensibility measurement, and how many patients received adjuvant radio- or chemotherapy.

**Table 2:** Patient characteristics between implant and mastectomy group

	Implant *n* = 16 (31.4%)	Mastectomy *n* = 35 (68.6%)	*p *value
Age in years (mean ± SD)	54.4 ± 6.4	52.2 ± 9.4	0.407
BMI in kg/m^2^ (mean ± SD)	25.0 ± 2.1	25.0 ± 3.1	0.936
Neuropathy due to chemotherapy	0 (0)	4 (11.4)	0.159
Number of previous breast surgeries	1.9 ± 1.0	1.2 ± 0.5	*0.001*
Time between mastectomy and sensibility measurement in months (median, IQR)	64 (20.5–100.5)	19.0 (12.5–30.0)	*0.025*
Radiotherapy	3 (18.8)	21 (60)	*0.006*
Chemotherapy	6 (37.5)	29 (82.9)	*0.001*

Data are shown as mean and standard deviation (SD), median and interquartile range (IQR), or absolute number and percentages, unless specified otherwise. None of the patients were smokers at the time of presentation

Statistically significant differences are indicated in italic

*BMI* body mass index

### Breast sensibility after mastectomy

A significantly higher monofilament index value was found in all nine measured areas of the breasts that underwent mastectomy (*n* = 35) compared to the contralateral, non-operated breasts, corresponding with less breast sensibility after mastectomy. The total monofilament index value of the mastectomy group was 3.84 (IQR 3.22–4.31) and the reference group was 2.44 (IQR 2.36–3.22), resulting in a *p* value of < 0.001.

### Breast sensibility after IBBR

The median monofilament index values with IQR were calculated for the women who underwent unilateral IBBR after mastectomy (*n* = 16). The total median monofilament index value of the implant group was 5.06 (IQR 4.20–5.32) and of their references 2.36 (IQR 2.36–2.83), indicating significantly impaired breast sensibility after IBBR (*p* < 0.001).

### Breast sensibility and factors of influence

The variables that were evaluated with simple linear regression were the number of previous breast surgeries, the time between mastectomy and sensory measurement, chemotherapy, radiotherapy, and IBBR. This showed a statistically significant association between IBBR and a higher difference in median monofilament values (*p* = 0.008). The statistically significant differences that are found in Table 3 were corrected using multivariable linear regression. None of the variables showed a statistically significant association with a higher difference in monofilament index values. IBBR nearly reached a significant association with impaired breast sensibility (*p* = 0.052), as shown in Table 3.

**Table 3:** Patient characteristics associated with the difference in median monofilament index values between the operated breasts and their references

	Univariable		Multivariable	
	β (SE)	*p *value	β (SE)	*p *value
Implant	0.897 (0.323)	*0.008*	0.843 (0.424)	0.052
Number of previous breast surgeries	0.294 (0.211)	0.171	0.138 (0.251)	0.585
Time between mastectomy and sensory measurement in months	0.001 (0.003)	0.721	− 0.004 (0.003)	0.259
Radiotherapy	− 0.490 (0.315)	0.126	0.033 (0.400)	0.935
Chemotherapy	− 0.628 (0.336)	0.067	− 0.375 (0.439)	0.397

Analysis performed with simple and multivariable linear regression analysis, statistically significant when *p* ≤ 0.05 (italic)

*β* beta coefficient, *SE* standard error

### Implant versus mastectomy

The independent samples *t*-test showed that the difference found in total median monofilament index values between the implant-based reconstructed breast and the breasts that underwent mastectomy alone was not significant in area 1 to 5. However, this difference was significant in the remaining areas, which were located on the nipple–areola complex (NAC), or where the NAC used to be. In addition, the total median monofilament index values of all areas were also significantly higher after IBBR (Table 4).

**Table 4:** Median monofilament index values per area measured in the mastectomy breasts and the breasts with an implant in situ

Median monofilament index value	Mastectomy (*n* = 35)	Implant (*n* = 16)	*p* value
1. Upper inner quadrant (UIQ)	3.22 (2.36–4.17)	3.61 (2.63–4.63)	0.120
2. Lower inner quadrant (LIQ)	3.61 (2.36–4.17)	3.84 (2.63–4.49)	0.359
3. Lower outer quadrant (LOQ)	3.61 (2.44–4.17)	4.38 (3.22–5.18)	0.053
4. Upper outer quadrant (UOQ)	3.61 (2.36–4.17)	4.01 (2.83–5.18)	0.166
5. Areola upper inner	4.17 (3.22–4.56)	4.53 (3.90–5.39)	0.184
6. Areola lower inner	4.08 (3.22–4.31)	5.32 (4.46–5.88)	*0.001*
7. Areola lower outer	4.08 (3.61–4.56)	5.46 (5.18–5.88)	< *0.001*
8. Areola upper outer	4.31 (3.22–4.93)	5.46 (4.25–5.88)	*0.020*
9. Nipple	4.31 (3.84–5.18)	5.46 (4.68–5.88)	*0.021*
10. Total breast	3.84 (3.22–4.31)	5.06 (4.13–5.39)	*0.012*

Data are shown as median with interquartile range (IQR)

The differences between the median index values per area are calculated, which was statistically significant in 4 of the 9 areas (italic)

### Sensibility best preserved

In the healthy breasts, the upper half of the breast seemed to be more sensitive than the lower half (*p* < 0.001), but we did not observe a significant difference between the sensation of the medial and lateral half (*p* = 0.612). This was confirmed by the analyses performed between the quadrants of which the *p* values are shown in Tables 5, 6, and 7. However, this was contradictory to what was found in the operated breasts (*n* = 51), where a significant lower monofilament index value was found in the medial part of the breast compared to those measured in the lateral part of the breast (*p* < 0.001), but no difference was found in mean monofilament values of the cranial and caudal parts (*p* = 0.207). In the mastectomy group, the same results were seen with a significant lower monofilament index value in the medial part of the breast compared to the lateral part, indicating better sensation medially. Again no difference was found between the cranial part and lower part of the mastectomy breasts (*p* = 0.965). After IBBR, the medial part of the breast was more sensitive than the lateral part (*p* = 0.011) and also the cranial part was more sensitive than the caudal part (*p* = 0.047).

**Table 5:** Mean monofilament index values of the medial half versus lateral half of the breast

	Medial	Lateral	*p* value	Cranial	Caudal	*p* value
Reference	2.48 (2.36–2.91)	2.40 (2.36–2.99)	0.612	2.38 (2.36–2.83)	2.50 (2.36–3.03)	< *0.001*
Operated	3.93 (3.18–4.48)	4.08 (3.38–4.89)	< *0.001*	3.93 (3.18–4.61)	4.03 (3.32–4.80)	0.207
Mastectomy	3.81 (2.83–4.32)	3.87 (3.32–4.37)	*0.006*	3.84 (3.06–4.46)	3.84 (3.01–4.22)	0.965
Implant	4.30 (3.60–4.87)	4.99 (3.98–5.61)	*0.011*	4.36 (3.68–5.10)	4.85 (4.05–5.27)	*0.047*

Data are shown as median with interquartile range (IQR)

Statistically significant differences are indicated in italic

**Table 6:** Mean monofilament index values of the medial quadrants versus the lateral quadrants of the breast

	UIQ	UOQ	*p* value	LIQ	LOQ	*p* value
Reference	2.36 (2.36–2.79)	2.36 (2.36–2.83)	0.508	2.40 (2.36–3.03)	2.44 (2.36–3.14)	0.901
Operated	3.95 (2.83–4.44)	4.01 (3.34–5.03)	*0.004*	3.96 (3.03–4.55)	4.12 (3.34–4.72)	*0.010*
Mastectomy	3.65 (2.83–4.44)	3.89 (3.27–4.55)	*0.023*	3.84 (2.79–4.31)	3.84 (3.22–4.83)	0.084
Implant	4.19 (3.61–4.96)	4.78 (3.57–5.45)	0.072	4.53 (3.92–4.76)	4.89 (4.21–5.61)	0.058

Data are shown as median with interquartile range (IQR)

Statistically significant differences are indicated in italic

**Table 7:** Mean monofilament index values of the cranial quadrants versus the caudal quadrants of the breast

	UIQ	LIQ	*p* value	UOQ	LOQ	*p* value
Reference	2.36 (2.36–2.79)	2.40 (2.36–3.03)	*0.002*	2.36 (2.36–2.83)	2.44 (2.36–3.14)	*0.017*
Operated	3.95 (2.83–4.44)	3.96 (3.03–4.55)	0.122	4.01 (3.34–5.03)	4.12 (3.34–4.72)	0.620
Mastectomy	3.65 (2.83–4.44)	3.84 (2.79–4.31)	0.510	3.89 (3.27–4.55)	3.84 (3.22–4.83)	0.650
Implant	4.19 (3.61–4.96)	4.53 (3.92–4.76)	0.159	4.78 (3.57–5.45)	4.89 (4.21–5.61)	0.185

Data are shown as median with interquartile range (IQR)

Statistically significant differences are indicated in italic`

### Clinical relevance

**Fig. 2:**
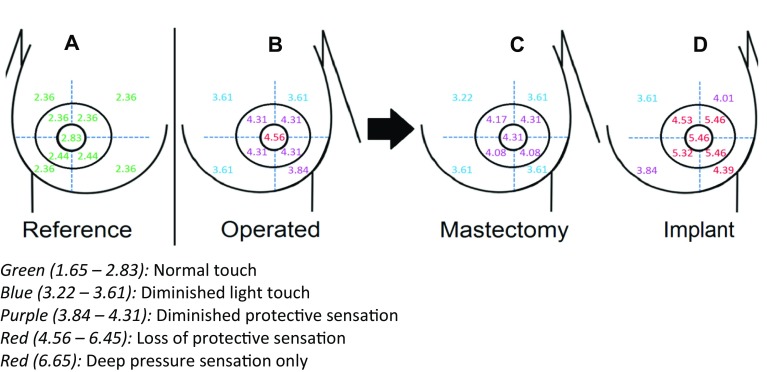
The median monofilament index values per area per (sub)group

The clinical relevance of a higher median monofilament value is shown in Fig. 2. It displays a visualization of the median monofilament index values per area in all operated breasts and the reference group (Fig. 2a, b). These are afterwards stratified into two groups: the women who only underwent mastectomy (Fig. 2c) and the women who additionally underwent implant-based breast reconstruction (Fig. 2d). Figure 2c shows diminished light touch in areas 1 to 4, diminished protective sensation in areas 5 to 8, and loss of protective sensation in area 9. Figure 2D shows similar results, but diminished light touch only in area 1, diminished protective sensation in areas 2 and 4, and loss of protective sensation in the remaining areas.

## Discussion

Although it is recognized that the breast sensibility is impaired after mastectomy or reconstruction, little attention is given to this particular aspect during preoperative counseling and patients are often not prepared for this unexpected sequel that negatively affects psychological well-being and the quality of life [9]. Women who underwent IBBR often state that their new breast feels different and describe them as hard, unyielding, lacking sensation, painful, or not feeling like their own [10]. In 1993, Peltonimi suggested for the first time that implants may have a negative influence on the sensation in the reconstructed breast [11]. However, to our knowledge this topic is yet to be studied properly. The available literature mainly focuses on sensation in the autologous reconstructed breast [12–16] and the influence of implants on breast sensibility after breast augmentation [17], rather than breast reconstruction.

The current study showed that the sensation in the operated breast is significantly impaired to that of a healthy non-operated breast. Five patients underwent surgery to the healthy breast before sensory measurement, which might impact sensation and affect comparison to the mastectomy breasts. Breast sensibility after reduction mammoplasty remains a controversial subject. Some studies suggest improvement of sensation after reduction mammoplasty in patients with gigantomastia [18] or at least retained in 80% [19], while others state impairment of breast sensibility after reduction mammoplasty [20, 21]. However, the maximum follow-up period in these studies was 6 months. Chiari concluded that breast sensibility returns to preoperative levels after approximately 6 to 12 months after mammoplasty [22]. This statement was confirmed by a literature review of Chiummariello [23]. The patients in our study were all more than 12 months after reduction or augmentation mammoplasty, suggesting normal sensation in the healthy breasts that served as control. This was confirmed by the results obtained by our sensory measurements. Therefore, we conclude that in the current study the contralateral breast surgeries were not affecting our results. Moreover, even if there would have been impaired sensation in the healthy breast, this would have amplified our results by providing a smaller difference in breast sensibility between the operated and healthy breasts. In addition, our results show that breast sensation following mastectomy is significantly better than breast sensation following implant-based breast reconstruction. Our hypothesis of why breast sensibility is more impaired after IBBR is tripartite: (1) The implant is a foreign body often placed partially beneath the major pectoral muscle. This obstructs profound innervation coming from underneath the major pectoral muscle and prevents neurotization of the skin. (2) The implant puts pressure on the remaining functional sensible nerves, pinching them, and causing pressure neuropathy. (3) In delayed breast reconstructions with implants, expansion of the skin envelope with a tissue expander is necessary for optimal fitting of the desired volume prosthesis. This reduces the amount of sensory receptors in the skin per square centimeter. In this study, no discrimination was made between immediate and delayed breast reconstruction with implants, because of the small number of patients. However, we assume that the majority of the patients with an implant underwent two-stage implant-based breast reconstruction, since a large prospective study in the Netherlands recently showed that one-stage implant-based breast reconstruction is associated with far more early postoperative complications compared to two-stage implant-based breast reconstruction [24]. The cutaneous sensory receptors are distributed and because neurotization of the skin is mechanically prevented by the implant, this consequently leads to impaired cutaneous sensibility of the breast. These are, however, still hypotheses and are yet to be studied properly with larger sample sizes and histological studies.

It is known for a long time that chemotherapy is associated with peripheral polyneuropathy, also known as chemotherapy-induced peripheral neuropathy (CIPN) [25]. This affects sensation in the distal extremities, limiting patients in their daily life. However, to our knowledge no previous study has suggested any association between chemotherapy and impaired breast sensibility. This is confirmed by the results of the current study. Moreover, if chemotherapy would have affected breast sensibility, the effect would be equal in both the operated and the contralateral healthy breast of the patient. Because the population in the current study functioned as their own control, this effect was considered irrelevant. Also, chemotherapy and radiotherapy were statistical significant baseline characteristics between the mastectomy and implant groups, which differed in favor of the mastectomy group. Therefore, the negative effect of IBBR on breast sensibility might even be underestimated and our hypothesis would be emphasized.

In 2007, Lagergren et al. concluded from their retrospective study that the sensibility was most affected in the upper part of the alloplastic reconstructed breast. They suggested that more extensive nerve damage is induced in the upper part of the breast by the operative technique, in which the dissections are more extended above the level of the reconstructed areola than below [26]. This is contradictory to our findings, in which the sensibility was best preserved in either the medial or cranial half of the alloplastic reconstructed breast. This might be explained by the anatomy of the sensible nerves of the breast. In the literature, there is still no consensus on the course of the sensible nerves of the breast. Some studies state that they run deep over the pectoral fascia and through the mammary gland [19, 27–30], others state that their course is superficial and close to the surface [31–33]. Since our results show a statistically significant better sensation in the medial part of all operated breasts, we hypothesize that the lateral branches of the medial intercostal nerves might have a superficial course close to the surface and that the anterior branches of the lateral intercostal nerves run through the mammary gland after they arise from the axillary line. Consequently, when performing a mastectomy or IBBR, the cutaneous branches of the lateral intercostal nerves might be sacrificed more often than those of the medial intercostal nerves. This might be of importance in autologous breast reconstructions considering the possibility to perform sensory nerve anastomosis.

The most important result is that, due to IBBR, breast sensation will be impaired to a level in which the cutaneous protective function is lost. This is important, because the patient is not able to react to pain stimuli. In addition, because the skin of the reconstructed breast is de-innervated, the skin is not able to react sufficiently to temperature changes, making the reconstructed breast prone to mechanical and thermal injuries [34–36].

We would like to stress that, even though these results are important and need to be brought to the attention of plastic and oncologic surgeons worldwide, we are not suggesting IBBR has to be banished. IBBR does have a positive effect on the QoL of breast cancer patients [37] and has to remain an option for patients seeking information on breast reconstruction. There is no technique that is suitable for all patients and there is no technique without its drawbacks. Autologous breast reconstruction is nowadays part of the standard care for breast cancer patients, but has the disadvantage of sensory alteration at the donor site, including a large scar. The patient has to be aware of all aspects of breast reconstruction, before making her decision. Breast sensibility has to become part of the preoperative counseling. In the end, it is always the patient’s choice.

## Conclusion

Statistically significant impairment concerning cutaneous threshold sensibility to touch after mastectomy compared to a healthy, non-operated breast was demonstrated, regardless of the time that has passed since the mastectomy. However, the cutaneous protective function is still intact, although it is impaired. When performing IBBR, the protective function is completely lost in nearly the entire breast. It is very important that this topic is brought to the attention of plastic and oncologic surgeons and that they inform their patients about this troublesome side effect and the associated risks. However, higher quality studies are required to generate more evidence and confirm these results.

## Electronic supplementary material

Below is the link to the electronic supplementary material.

Supplementary material 1 (PDF 302 KB)

Supplementary material 2 (PDF 415 KB)

## Data Availability

Original data are available as supporting information.
