# Supplementary material for: Breast sensibility after mastectomy and implant-based breast reconstruction
Source: Breast Cancer Res Treat. 2019 Feb 12;175(2):369–78. doi: 10.1007/s10549-019-05137-8 (PMC6533215; doi:10.1007/s10549-019-05137-8)
Supplement: Supplementary file 1 — Supplementary material 1 (PDF 302 KB) [file 10549_2019_5137_MOESM1_ESM.pdf]

ID	Age	Neuro-logical	Remarks	Previous breast surgeries	Reason of previous breast surgery	Implant	Radio-therapy	Chemo-therapy	Months since mastectomy	BMI	Smoker
1	59	0		1		0	1	1	1	29,69	0
2	62	0		1		0	1	1	20	27,77	0
3	59	0		3	1x mastectomy with tissue expander; 2x capsulotomy	1	1	0	296	26,08	0
4	52	0		1		0	0	1	69	23,26	0
5	57	0		1		0	1	1	29	24,77	0
6	33	0		1		0	1	1	11	19,84	0
7	42	0		1		0	0	0	3	25,35	0
8	65	0		1		0		1	19	25,71	0
9	57	0		2	1x mastectomy with tissue expander; 1x placement definitive implant	1	0	0	67	27,69	0
10	45	0		1		0	1	1	21	24,84	0
11	51	0		1		0	1	1	17	26,83	0
12	49	0		1		0	0	1	58	28,73	0
13	61	0		2	1x mastectomy with tissue expander; 1x placement definitive implant	1	0	0	322	25,51	0
14	53	1	Polyneuropathy due to chemo	2	1x mastectomy with tissue expander; 1x explantation tissue expander	0	0	1	11	29,32	0
16	57	0		2	1x mastectomy with tissue expander; 1x placement definitive implant	1	0	0	73	24,51	0
17	45	0		1		0	0	0	59	22,4	0
18	66	1	Polyneuropathy due to chemo	2	1x mastectomy	0	1	1	76	31,64	0
19	43	1	Polyneuropathy due to chemo	1		0	1	1	36	31,28	0
20	37	0		1		0	1	1	15	21,48	0
21	47	0		1		0	0	1	3	28,41	0
22	69	0		2	1x breast conserving therapy; 1x mastectomy with direct alloplastic reconstruction	1	0	0	108	28,06	0
23	55	0		2	1x mastecomy with direct alloplastic reconstruction; 1x explantation implant	0	0	1	24	22,4	0
24	55	0		2	1x mastectomy with tissue expander; 1x placement definitive implant	1	0	0	72	24,57	0
25	51	0		1	1x mastectomy with direct alloplastic implant	1			19	24,49	0
26	56	0		2	1x mastectomy with tissue expander; 1x placement definitive implant	1	0	0	80	24,54	0
27	48	0		3	1x breast conserving therapy; 1x mastectomy with tissue expander; 1x explantation tissue expander	0	0	1	37	22,77	0
28	39	0		1		0	1	1	99	24,22	0
29	54	0		2	1x breast conserving therapy; 1x mastectomy	0	0	0	21	20,06	0
30	66	0		1		0	0	0	9	26,13	0
31	68	0		1		0	0	1	16	22,48	0
32	50	0		2	1x mastectomy with direct alloplastic implant	1	1	1	16	23,67	0
33	54	0		1		0	1	1	22	29,41	0
34	48	0		2	1x mastectomy with tissue expander; 1x placement definitive implant	1	1	1	21	24,09	0
36	48	0		1		0	1	1	3	25,51	0
37	42	0		1	1x mastectomy with tissue expander	1		1	11	26,87	0

38	48	0		1	1x mastectomy with tissue expander	1	0	0	16	25,1	0
39	58	0		2	1x mastectomy with direct alloplastic reconstruction; 1x explantation implant	0	0	0	7	24	0
40	52	0		1		0	1	1	15	21,26	0
41	51	0		1		0	1	1	9	21,48	0
42	41	0		1		0	1	1	11	24,86	0
43	49	0		1		0	0	0	9	23,32	0
44	34	0		1		0	0	1	12	23,8	0
45	63	1	Polyneuropathy due to chemo	1		0	0	1	105	21,23	0
47	55	0		2	1x breast augmentation; 1x mastectomy with direct alloplastic reconstruction	1		1	46	26,3	0
48	62	0		1		0	1	1	29	25,15	0
49	51	0		1	1x mastectomy with tissue expander	1	0	0	36	28,08	0
50	57	0		1		0		1	24	24,68	0
51	60	0		2	1x mastectomy with direct alloplastic reconstruction; 1x replacement of implant	1	0	1	126	20,98	0
52	51	0		5	several breast reconstructive surgeries	1	0	1	61	21,05	0
53	63	0		1		0	1	1	31	25,28	0
54	59	0		1		0	1	1	17	26,64	0
