# Supplementary material for: Breast sensibility after mastectomy and implant-based breast reconstruction
Source: Breast Cancer Res Treat. 2019 Feb 12;175(2):369–78. doi: 10.1007/s10549-019-05137-8 (PMC6533215; doi:10.1007/s10549-019-05137-8)
Supplement: Supplementary file 2 — Supplementary material 2 (PDF 415 KB) [file 10549_2019_5137_MOESM2_ESM.pdf]

ID		1	2	3	4	5	6	7	8	9
**1**	**Right**	4,08	4,08	4,08	3,22	4,08	4,08	4,08	4,08	4,74
	**Left**	3,22	3,61	3,61	3,61	3,22	3,22	3,22	3,22	3,22
**2**	**Right**	2,44	2,36	2,44	2,36	2,83	2,36	2,83	0,236	2,83
	**Left**	3,22	3,61	3,84	3,61	4,08	4,31	3,61	4,17	4,17
**3**	**Right**	2,36	2,36	2,36	2,36	2,36	2,83	2,36	2,36	2,83
	**Left**	5,88	5,88	5,18	5,88	5,88	5,88	5,88	5,88	5,88
**4**	**Right**	3,22	3,22	3,61	3,61	2,83	2,83	4,08	2,83	3,84
	**Left**	3,84	3,61	3,22	3,84	3,22	3,22	3,22	3,22	3,22
**5**	**Right**	2,83	2,36	2,36	2,83	2,83	2,83	2,83	2,83	3,22
	**Left**	4,17	5,07	4,31	5,07	4,93	5,18	5,18	5,88	6,65
**6**	**Right**	2,36	2,36	2,36	2,36	2,36	2,36	2,36	2,36	2,36
	**Left**	2,83	2,36	2,44	2,83	2,83	3,22	3,61	2,83	3,22
**7**	**Right**	2,83	3,22	4,31	5,18	3,61	3,22	4,17	4,31	4,56
	**Left**	2,36	2,36	2,36	2,36	2,36	2,36	2,36	2,36	2,36
**8**	**Right**	2,36	2,36	2,36	2,36	2,44	2,44	2,44	2,44	2,44
	**Left**	2,36	2,44	2,44	2,44	2,83	2,83	4,08	4,08	4,08
**9**	**Right**	3,61	3,84	6,1	4,56	4,17	5,18	6,1	5,88	6,1
	**Left**	2,36	2,36	2,36	2,36	2,36	2,44	2,83	2,36	3,22
**10**	**Right**	2,36	2,36	2,36	2,36	2,36	2,36	2,36	2,36	3,22
	**Left**	2,36	2,36	2,36	3,22	3,84	3,22	3,84	3,84	4,17
**11**	**Right**	3,61	3,61	3,61	3,61	5,46	4,31	4,17	4,93	5,46
	**Left**	2,36	2,36	2,36	2,36	2,36	2,83	2,83	2,36	2,83
**12**	**Right**	2,36	2,36	2,36	2,36	2,44	2,44	2,44	2,44	2,83
	**Left**	2,36	2,36	2,44	2,36	2,44	2,36	2,44	2,36	2,44
**13**	**Right**	2,36	2,36	2,44	2,36	2,36	2,36	2,36	2,36	2,44
	**Left**	2,36	2,36	2,44	2,36	2,36	2,36	2,36	2,36	2,44
**14**	**Right**	4,56	3,84	4,17	4,17	4,56	4,31	4,56	4,56	4,56
	**Left**	2,83	3,22	3,22	3,22	3,61	3,61	3,61	3,61	3,61
**16**	**Right**	3,22	3,61	3,22	3,61	3,22	3,22	3,22	3,22	3,22
	**Left**	3,61	3,22	3,22	2,83	4,08	4,08	4,08	4,08	4,08
**17**	**Right**	2,36	2,36	2,36	2,36	2,44	2,44	2,44	2,44	2,44
	**Left**	4,08	4,56	4,08	2,83	4,31	4,31	4,31	4,31	4,31
**18**	**Right**	2,36	2,36	2,83	2,36	2,83	2,83	2,83	2,83	2,83
	**Left**	4,31	4,08	3,84	4,08	4,56	4,56	4,31	4,56	4,56
**19**	**Right**	2,36	2,36	2,36	2,36	2,36	2,36	2,36	2,36	2,83
	**Left**	2,83	2,83	3,22	3,84	2,83	2,83	3,22	3,22	4,31
**20**	**Right**	2,83	3,22	3,22	2,83	3,61	3,61	3,61	3,61	3,61
	**Left**	2,36	2,36	2,36	2,36	2,36	2,36	2,36	2,36	2,36
**21**	**Right**	4,56	5,07	6,1	2,83	5,46	5,88	6,1	5,18	6,1
	**Left**	2,36	2,83	3,22	2,36	3,22	3,22	3,22	3,22	3,22
**22**	**Right**	3,84	3,84	3,84	3,84	4,74	5,18	5,18	4,74	5,88
	**Left**	2,36	2,36	2,44	2,36	2,36	2,36	2,36	2,44	2,83
**23**	**Right**	2,36	2,36	2,36	2,36	2,36	2,36	2,36	2,36	2,36
	**Left**	2,83	4,17	4,17	2,83	4,17	4,17	4,17	4,17	4,31
**24**	**Right**	2,44	2,36	2,83	2,36	5,46	5,46	5,46	5,46	5,46
	**Left**	2,36	2,36	2,36	2,36	2,36	2,36	2,36	2,36	2,83
**25**	**Right**	4,08	4,08	5,88	5,18	4,31	5,46	5,88	5,88	5,88
	**Left**	2,36	2,36	2,83	2,36	2,36	2,83	2,83	2,83	2,83
**26**	**Right**	3,22	2,44	3,22	3,22	3,84	3,61	6,22	3,22	3,84
	**Left**	2,36	2,83	2,44	2,83	3,84	3,84	3,84	2,36	2,83
**27**	**Right**	2,44	2,83	2,83	2,44	2,83	3,22	3,22	2,83	3,22

	**Left**	3,61	3,61	3,61	3,61	3,84	4,17	4,08	4,31	4,31
**28**	**Right**	2,36	2,36	2,36	2,36	2,36	2,36	2,36	2,36	2,36
	**Left**	2,36	2,36	2,83	4,08	4,17	3,84	3,84	4,08	4,08
**29**	**Right**	2,36	2,36	2,36	2,36	3,22	3,22	4,08	4,17	3,84
	**Left**	2,36	2,36	2,36	2,36	2,36	2,36	2,36	2,36	2,36
**30**	**Right**	4,31	4,31	4,31	4,31	4,31	4,31	4,31	4,31	4,31
	**Left**	4,31	4,31	4,31	4,31	4,31	4,31	4,31	4,31	4,31
**31**	**Right**	3,22	3,22	3,84	3,22	2,44	2,44	2,44	2,44	2,44
	**Left**	4,31	4,31	4,31	5,18	4,31	4,08	4,08	5,07	3,84
**32**	**Right**	2,36	2,36	2,36	2,36	2,36	2,36	2,36	2,36	2,36
	**Left**	4,31	3,61	5,18	3,61	4,31	4,31	6,1	5,46	6,1
**33**	**Right**	2,44	2,83	3,22	2,83	3,22	3,22	3,22	3,22	3,22
	**Left**	2,36	3,84	3,84	4,17	4,56	3,84	3,84	4,93	4,93
**34**	**Right**	2,36	2,36	2,36	2,36	2,36	0,588	5,88	2,36	5,88
	**Left**	2,36	2,36	2,36	2,36	2,36	2,36	2,36	2,36	2,36
**36**	**Right**	2,36	2,36	2,36	2,36	3,22	3,22	3,22	3,22	3,22
	**Left**	2,36	2,36	2,36	2,36	2,36	2,36	2,36	2,36	2,36
**37**	**Right**	2,36	2,36	2,36	2,36	2,36	3,22	2,83	2,36	3,22
	**Left**	3,61	3,22	3,61	2,83	5,46	5,88	5,46	5,18	5,46
**38**	**Right**	5,46	3,84	5,88	4,17	5,18	5,46	5,88	5,88	5,88
	**Left**	2,36	2,36	2,36	2,36	2,44	2,44	2,44	2,44	2,83
**39**	**Right**	2,36	2,36	2,36	2,36	2,36	2,36	2,36	2,36	2,83
	**Left**	2,36	2,36	2,36	2,36	3,22	5,18	5,18	4,31	5,18
**40**	**Right**	2,36	2,36	3,84	3,84	3,22	3,22	3,84	3,84	3,84
	**Left**	2,36	2,36	2,36	2,36	2,36	2,36	2,36	2,36	2,36
**41**	**Right**	2,36	2,36	2,36	2,36	2,36	2,36	2,36	2,36	2,36
	**Left**	4,17	4,17	4,17	4,17	4,93	4,93	4,56	5,18	5,18
**42**	**Right**	2,36	2,36	2,36	2,36	2,36	2,36	2,36	2,36	2,83
	**Left**	4,17	3,61	3,61	3,84	5,07	3,84	4,93	4,31	5,88
**43**	**Right**	3,22	4,31	3,22	5,07	5,07	5,46	4,93	5,18	5,46
	**Left**	2,36	2,36	2,36	2,36	2,36	2,36	2,36	2,36	2,36
**44**	**Right**	2,36	2,36	2,36	2,36	2,36	2,36	2,36	2,36	2,36
	**Left**	3,61	2,36	2,36	2,36	4,31	3,61	3,61	4,93	4,93
**45**	**Right**	2,36	2,36	3,84	2,36	2,36	2,36	2,36	2,36	4,93
	**Left**	2,36	2,36	2,44	2,36	2,44	2,44	2,36	2,36	2,36
**47**	**Right**	5,07	5,07	4,93	6,45	5,18	5,88	5,18	6,45	5,18
	**Left**	2,36	4,13	3,22	2,36	3,22	3,84	3,84	3,84	3,22
**48**	**Right**	2,44	3,84	3,61	2,36	3,22	3,22	4,17	2,36	4,08
	**Left**	2,44	2,83	2,83	2,36	2,83	3,61	2,83	2,83	2,36
**49**	**Right**	2,36	4,56	4,93	5,18	2,36	4,93	5,18	5,18	5,18
	**Left**	2,36	2,36	2,36	2,36	2,36	2,36	2,36	2,36	2,36
**50**	**Right**	4,31	4,56	4,74	4,17	4,56	5,18	5,18	5,46	5,46
	**Left**	2,44	2,83	2,83	2,44	2,44	2,83	2,83	2,36	2,36
**51**	**Right**	3,61	4,74	3,61	4,74	4,74	6,1	5,18	6,1	5,07
	**Left**	2,36	2,36	2,36	2,36	3,61	3,61	3,61	3,61	3,22
**52**	**Right**	2,36	2,36	2,36	2,36	2,36	2,36	2,36	2,36	3,22
	**Left**	4,74	4,31	5,18	5,46	5,46	5,07	5,46	5,46	4,56
**53**	**Right**	4,74	5,07	5,07	6,10	6,10	6,10	6,10	6,10	5,88
	**Left**	2,36	2,36	2,36	2,36	2,36	3,84	2,36	3,84	3,84
**54**	**Right**	2,44	3,84	3,84	2,44	2,83	3,61	3,61	3,84	3,22
	**Left**	4,56	3,84	2,83	4,74	4,31	4,08	3,84	5,46	4,17
